# Unveiling an Immunological Mystery: Deciphering the Durability Divide in Vaccine-Elicited Antibody Responses

**DOI:** 10.2174/011570162X366336250707084941

**Published:** 2026

**Authors:** George K. Lewis, Stanca Ciupe, Mohammad Sajadi

**Affiliations:** 1 Division of Vaccine Research, Institute of Human Virology, Department of Microbiology and Immunology, University of Maryland School of Medicine, Baltimore, Maryland 21201, United States; 2 Department of Mathematics, Virginia Polytechnic Institute and State University, 225 Stanger Street, Blacksburg, 24060, VA, United States; 3 Division of Clinical Care and Research, Institute of Human Virology, Department of Medicine, University of Maryland School of Medicine, Baltimore, Maryland, 21201, United States

**Keywords:** Antibody durability, HIV, Vaccine, broadly neutralizing antibodies, long-lived plasma cells, CD21, antibody secretion rates, epitope multivalency, T-bet+ CD21 low Antibody Secreting Cells

## Abstract

Achieving durable antibody-mediated protection remains critical in vaccine development, particularly for viral diseases like COVID-19 and HIV. We discuss factors influencing antibody durability, highlighting the role of long-lived plasma cells (LLPCs) in the bone marrow, which are essential for sustained antibody production over many years. The frequencies and properties of bone marrow LLPC are critical determinants of the broad spectrum of antibody durability for different vaccines. Vaccines for diseases like measles and mumps elicit long-lasting antibodies; those for COVID-19 and HIV do not. High epitope densities in the vaccine are known to favor antibody durability, but we discuss three underappreciated variables that also play a role in long-lived antibody responses. First, in addition to high epitope densities, we discuss the importance of CD21 as a critical determinant of antibody durability. CD21 is a B cell antigen receptor (BCR) complex component. It significantly affects BCR signaling strength in a way essential for generating LLPC in the bone marrow. Second, all antibody-secreting cells (ASC) are not created equal. There is a four-log range of antibody secretion rates, and we propose epigenetic imprinting of different rates on ASC, including LLPC, as a factor in antibody durability. Third, antibody durability afforded by bone marrow LLPC is independent of continuous antigenic stimulation. By contrast, tissue-resident T-bet+CD21low ASC also persists in secondary lymphoid tissues and continuously produces antibodies depending on persisting antigen and the tissue microenvironment. We discuss these variables in the context of making an HIV vaccine that elicits broadly neutralizing antibodies against HIV that persist at protective levels without continuous vaccination over many years.

## 1. INTRODUCTION

Achieving durable antibody-mediated protection remains a significant goal in vaccine development, with no specific approaches known to elicit long-lasting protection against infectious diseases reliably. This problem is especially acute in developing vaccines against viral diseases, exemplified by the recent COVID-19 pandemic. The historical development and deployment of multiple COVID-19 vaccines in less than two years is an unequaled achievement across all levels of biomedical science, industry, and government (reviewed in [1-4]). The COVID-19 vaccines currently licensed in the United States elicit protection against SARS-CoV-2-associated disease and mortality that appears durable at the time of writing, while antibodies capable of blocking infection appear short-lived [5]. Rapidly waning protection against SARS-CoV-2 infection follows the pattern of seasonal coronaviruses [6-10]. A human reinfection study of sixteen volunteers challenged with the 229E seasonal coronavirus showed that neutralizing antibodies peaked at three weeks and waned close to background by fifty-two weeks when six of nine volunteers were infected after a second virus challenge [10]. While antibody escape mutations favor coronavirus reinfections, the homologous 229E human challenge study shows that poor antibody durability is also at play. The short durability of protection elicited by coronavirus infection starkly contrasts with infections with other non-persisting viruses. For example, a single infection with measles or mumps elicits antibody responses that persist with a half-life of approximately two hundred years [11]. The reasons underlying the broad spectrum of antibody durability for different vaccines are unknown. However, it is known that a unique population of long-lived plasma cells in the bone marrow is responsible for "serological memory" that is maintained without antigen stimulation long after infection or vaccination (reviewed in [12-15]). In this report, we propose that the ability of vaccines to elicit this plasma cell population is the primary factor underlying the durability of antibody-mediated protection. In this light, the antibody durability problem is an unsolved concern for developing vaccines against HIV that elicit broadly neutralizing antibodies (bnAbs) (reviewed in [16,17]). Accordingly, we will focus on the origin of bnAbs in people living with HIV (PLWH) with emphasis on plasma cell subsets in the bone marrow and the impact on HIV vaccine candidates under development. Further, we will frame the problem with broader perspectives on how HIV vaccine studies can be exploited to solve the antibody durability problem for other vaccines. In this regard, we will discuss the impact of recent studies of bone marrow plasma cell populations in SARS-CoV-2 infection and vaccination, coupled with the development of mathematical models to analyze and predict antibody durability after vaccination against HIV.

## 2. STUDY METHODOLOGY

We developed this review using several complementary approaches to explore antibody durability and long-lived plasma cell responses. First, we reviewed the scientific literature, including both classic studies and recent findings in immunology, virology, and vaccine research. We performed standard Boolean searches using the PubMed portal and Google Scholar to query the National Library of Medicine's literature database, which comprises over 38 million citations. Data from clinical trials, longitudinal studies, and experimental models were analyzed to understand how antibodies and plasma cells respond over time after vaccination or infection. We compared immune responses across different vaccines, infections, and experimental conditions, focusing on factors such as epitope multivalency, CD21 signaling, and adjuvant effects. Findings from both animal and human studies were integrated using data from techniques like flow cytometry, ELISPOT, and single-cell analysis. Based on these findings, we proposed new hypotheses, including how CD21 may influence antibody durability and how multivalent antigens may contribute to the generation of long-lived plasma cells. By combining insights from multiple scientific fields, the review highlights key concepts, identifies gaps in current knowledge, and suggests directions for future research.

### 2.1. Durability of Vaccine Elicited Responses with Antibody Correlates of Protection

The seminal 2007 NEJM report from Amanna, Carlson, and Slifka [11] sets the stage for our discussion. In that study, the authors carried out longitudinal analyses of antibody responses against viruses and bacterial toxoids (tetanus and diphtheria) of forty-five volunteers for whom banked sera were available, covering twenty-six years of employment at the Oregon Regional Primate Center. Memory B cell (B_Mem_) frequencies were determined for forty volunteers by limiting dilution analysis for each antigen. Four key findings emerged from the study mentioned above [11]. First, anti-viral antibody responses after infection can exhibit exceptional durability, with estimated half-lives ranging from approximately 50 years for varicella-zoster to over 200 years for viruses such as measles and mumps. Conversely, antibody responses to non-replicating antigens, such as those of tetanus and diphtheria, display considerably shorter half-lives of 11 and 19 years, respectively. Second, there was no significant association between circulating B_Mem_ frequencies and antibody titers for most of the responses studied. This observation is consistent with the independent regulation of B_Mem_ and long-lived antibody-secreting plasma cells, each contributing distinctively to maintaining long-term protective immunity. Third, repeated infection or vaccination does not significantly increase antibody durability. This observation also supports the conclusion that B_Mem_ and long-lived plasma cells are independently regulated. Fourth, antigen intrinsic properties can significantly influence the duration of humoral immunity, where multivalent viruses favor durability. This study raises the question of how these findings extend to other vaccines in more recent studies.

**Fig. (1).**
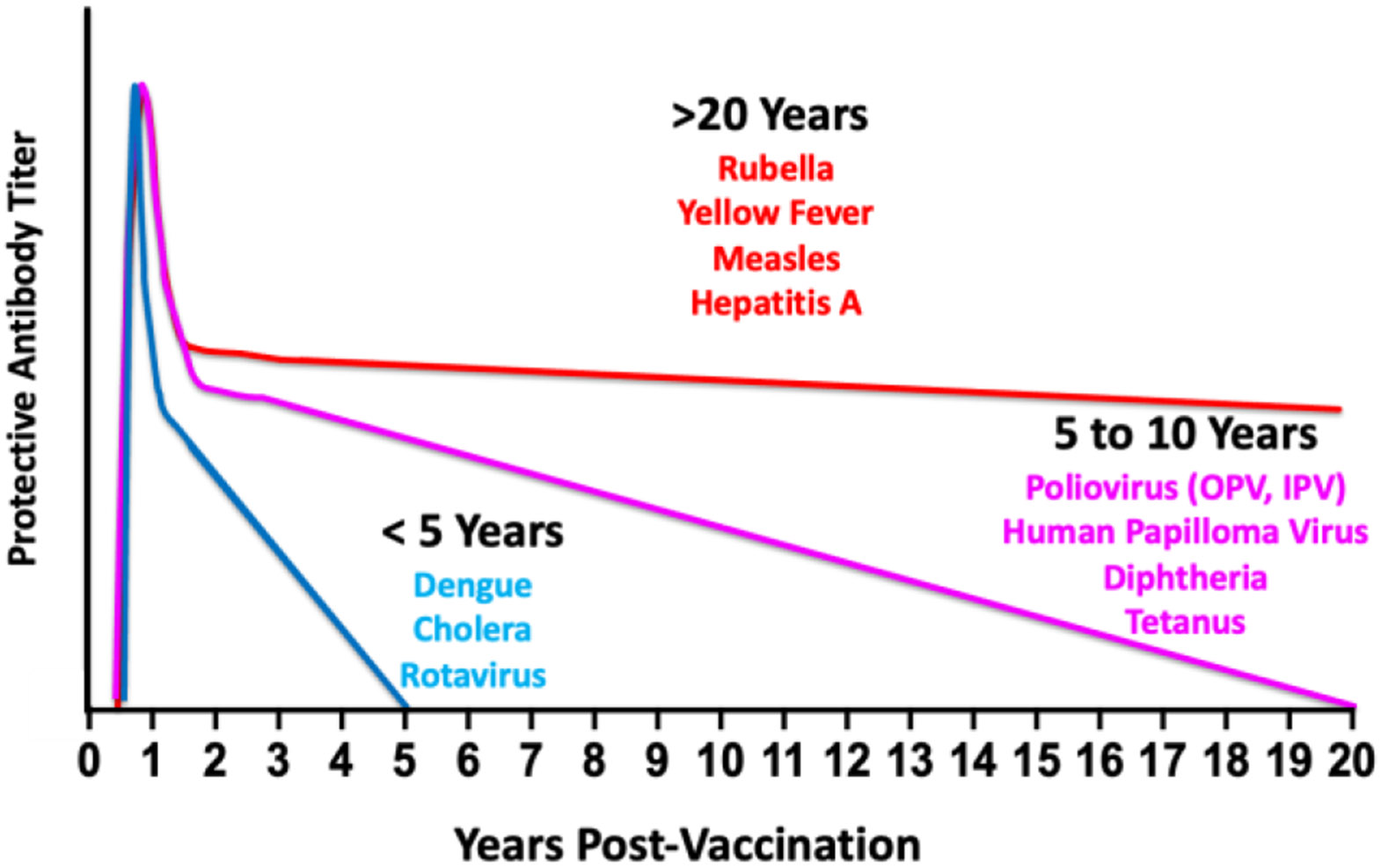
Patterns of Protective Antibody Durability This figure schematically depicts the three patterns of antibody durability discussed in [18] Several representative vaccines are shown to illustrate the three patterns: >20 years, 5 to 20 years, and < 5 years.

This question is framed in an excellent recent review that surveyed the durability of protection for thirty-four licensed vaccines against infectious diseases [18]. Three patterns of durability emerged from that analysis (our discussion is limited to vaccines with antibody correlates), as shown schematically in Fig. (1). The first pattern comprises vaccines that elicit protection lasting more than twenty years. Vaccines against rubella, measles, yellow fever, and hepatitis A are shown as examples in Figure 1 (the reader is referred to Table 1 in [18] for a full listing of the vaccines). The second pattern comprises vaccines that elicit five to twenty years of protection. These include vaccines against poliovirus (both inactivated and live attenuated polio vaccines), human papillomavirus, diphtheria, and tetanus. The third pattern comprises vaccines that elicit protection lasting less than five years. These include vaccines against the dengue virus, rotavirus, and cholera. Interestingly, no single property of the vaccines discussed in [18] predicts the durability of protection. For example, the vaccines against rubella, measles, and yellow fever are attenuated replication-competent viruses, whereas the vaccine against hepatitis A is inactivated. All four vaccines elicit protection lasting more than twenty years. The vaccines that elicit protection lasting between five and twenty years present a similar picture. One vaccine against polio is a replication-competent attenuated virus, and the other is inactivated.

The vaccine against human papillomavirus is an empty viral capsid, whereas the vaccines against tetanus and diphtheria are inactivated toxins. Finally, the vaccines that elicit protection lasting less than five years are all replication-competent attenuated viruses. While the authors listed many variables that might affect the durability of protection [18], no single immunogen form or adjuvant formulation reliably elicits durable protective antibody responses. At first glance, this might disfavor the hypothesis that infection with multimeric viruses favors longer durability. However, the most durable protection of the vaccines listed in [18] is multivalent particulate antigens such as virus particles and protein oligomers (c.f. [18]). These considerations suggest that antigenic multivalency is necessary but not always sufficient for optimally durable antibody responses. Studies in small animal models support this hypothesis and offer clues about additional biological variables required for multivalent antigens to elicit long-lived antibody responses.

### 2.2. Epitope Multivalency and Antibody Durability

For over fifty years, it has been known that epitope multivalency strongly influences an immunogen's ability to elicit antibody responses [19-23]. For most of those years, the conventional view was that epitope multivalency influences the number of B cell antigen receptors (BCR) cross-linked by the immunogen on the B cell surface and the strength of signaling via the BCR. A series of classic studies using mathematical modeling and an immunogen with haptens on a flexible polyacrylamide backbone suggested that immunogenicity is quantized with a minimum of twelve to sixteen BCR being continuously joined spatially as a cluster on the B cell surface to initiate an antibody response [23-25]. This observation contrasted with "minimal immunogens" studies, where antibody responses can be elicited by simple synthetic immunogens comprised of a single B cell epitope coupled with a single T cell epitope [26, 27]. This paradox has been resolved by more recent studies showing that BCRs are clustered into membrane microdomains on the B cell surface and are autoinhibited in their steady state (reviewed in [28]) and that even monovalent epitopes can dissociate BCRs to release the autoinhibition and initiate BCR signaling [28, 29]. Even in this model of BCR activation, epitope multivalency elicits distinct patterns of signaling that favor immunogenicity [28]. Regardless of the BCR to sense and respond to monovalent epitopes, recent studies show that epitope multivalency plays a role in eliciting durable antibody responses.

For example, immunization of mice with a novel multilamellar lipid nanoparticle [30]displaying multiple copies of the VMP001 circumsporozoite antigen (CSP) from *Plasmodium vivax* on their surface dramatically increased the durability of the antibody response to VMP001 as compared with immunization with monomeric VMP001 [31]. This result is striking because many previous studies found that while immunization with CSP antigens can protect against malaria in animal models, the protective antibody responses are very short-lived. This is also the case for the WHO-licensed RTS, S malaria vaccine optimized for immunogenicity via fusing the CSP fragment with the hepatitis B surface antigen and formulation with potent AS01E adjuvant (reviewed in [32]). In a four-year efficacy study of RTS, AS01E in children of malaria-endemic African countries, vaccine efficacy was 43.6% in the first year post-vaccination; however, vaccine efficacy fell to −0.4% by year four of the study [33]. While vaccine factors other than multivalency complicate this result, the results in mice using multivalent VMP001 [31] suggest that increasing epitope multivalency might prolong the protection elicited by next-generation malaria vaccines.

**Fig. (2).**
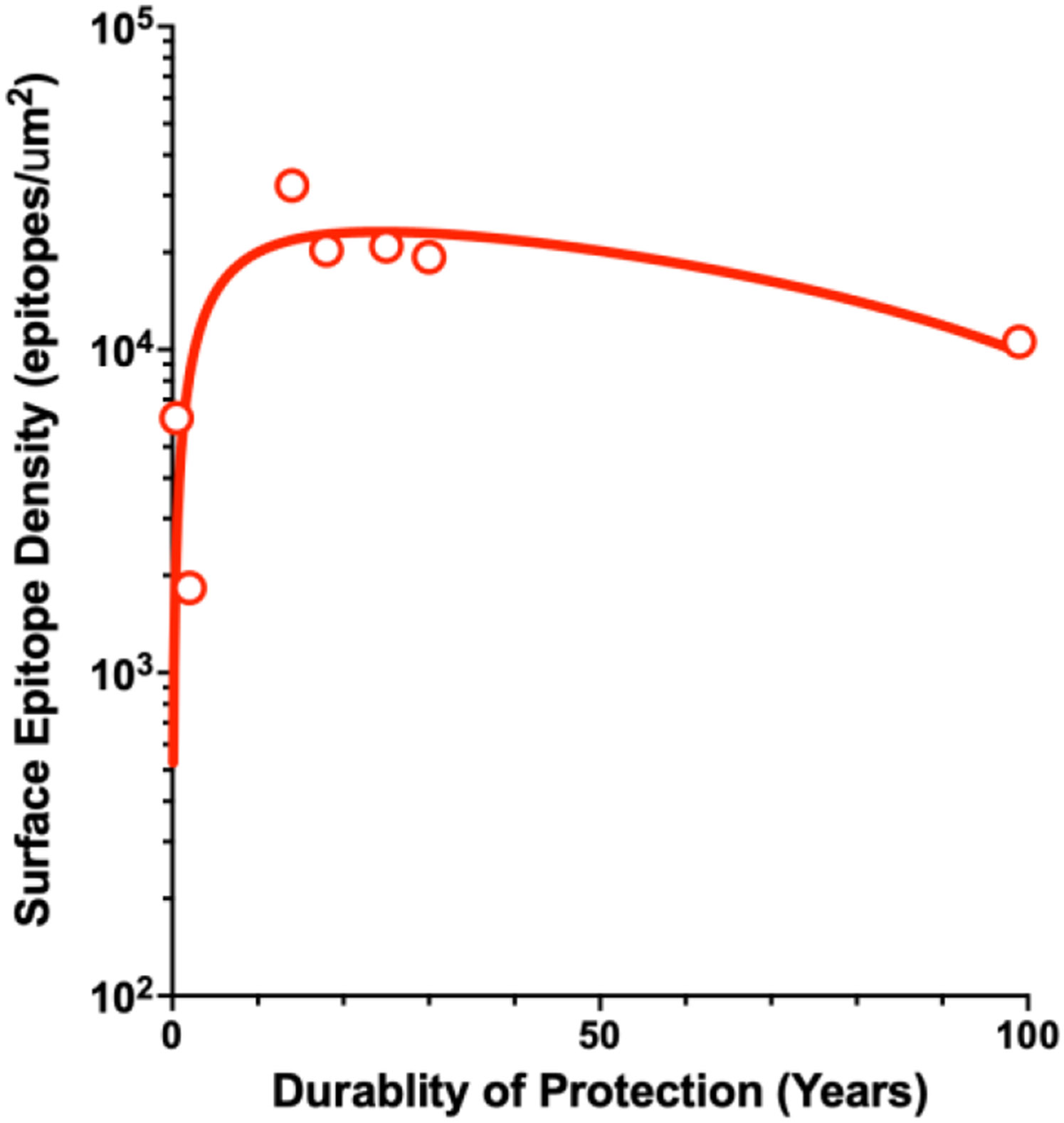
Relationship Between Surface Epitope Densities and Durability of Antibody-dependent Protection. The relationship between epitope densities on seven viruses (rotavirus, influenza, yellow fever, poliovirus, hepatitis B, hepatitis A, and human papilloma virus) and the durability of antibody-dependent protection is shown for their respective vaccines. Epitope densities were estimated from Fig. (**4**) in [38] using the Digitizelt software package. Protection durability values were from Table **1** in [18]. Non-linear curve fitting was performed using the GraphPad Prism software package.

More recent systematic studies using model antigens arrayed on virion-sized liposome surfaces have addressed the role of epitope multivalency in antibody durability [34-37] based on "the density code" proposed in [38] where it was recognized that epitope densities on eight viruses with licensed vaccines and HIV vary by approximately two orders of magnitude. Using the durability data from Table **1** in [18] and the virus surface epitope density data from Figure [38], Fig. (2) shows the relationship between the durability of protection and virus surface epitope density for seven licensed vaccines. A threshold epitope density greater than ten thousand determinants per cubic micron correlates with more than five years of protection. While this analysis is very preliminary, the epitope density concept is supported by elegant experiments [34-37, 39, 40] in mice using virus-sized (120 nm diameter) liposomal nanoparticles conjugated with protein antigens with defined average spatial densities corresponding to circulating viruses [38]. Compared with the monovalent model antigens (hen egg white lysozyme (HEL) and the RBD domain of SARS-CoV-2 (RB), a single inoculation of HEL or RBD conjugated to the virus-sized nanoparticles elicited antibody responses that lasted over six hundred days, which is close to the average lifespan of a mouse. Robust antibody affinity maturation was also observed, and it appears that BCR activation was influenced more by epitope densities than initial binding affinities [35], as suggested by a recent theoretical analysis [41]. Interestingly, including nucleic acid adjuvants in the particles increased response magnitude but did not affect the durability. These results formally demonstrate a relationship between antibody durability and epitope multivalency.

The preceding findings on epitope multivalency and antibody durability are consistent with the "multivalency and antigenic threshold" model of protective antibody responses proposed by Slifka and Amana [42], which extends on their imprinted lifespan model of antibody durability [43]. In this model, BCR signaling strength is a crucial determinant of plasma cell lifespan that is epigenetically imprinted during B cell activation. Earlier studies in mice showed that BCR signaling strength determines B cell fate [44], and the imprinted lifespan model proposed that multivalent epitopes presented such that they optimally elicit BCR signaling skew B cell fate toward the production of long-lived plasma cells. This raises the question of how BCR signaling complexes affect the imprinting of plasma cell lifespans.

### 2.3. The Underappreciated Role of CD21 in Antibody Durability

The details of BCR signaling strength are beyond this review's scope; however, one aspect of BCR signaling has escaped prior discussions of antibody durability [16, 17, 42, 45]: the essential role of CD21 in long-lived antibody responses [46-48]. CD21 is a coreceptor with CD81 in the BCR signaling complex ([49] and reviewed in [50]), which lengthens the duration of BCR signaling [51,52], amplifying signaling strength [52, 53]. CD21, also called complement receptor 2 (CR2), binds the C3d fragment of C3 and serves as the principal receptor for Epstein-Barr Virus (EBV), reviewed in [54, 55]. CD21 is also expressed on follicular dendritic cells, enabling antigen retention and presentation via C3d-associated antigen [54, 55]. Given the multifaceted role of CD21 and C3d in the immune response (reviewed in [54-57]), numerous studies showed that incorporating C3d into vaccine immunogens enhances immunogenicity ([58-60] and reviewed in [61]). However, none of these studies systematically addressed the effect of C3d on antibody durability. The connection between CD21 and antibody durability comes from studies using CD21 knockout mice [46, 48, 62-64]. The first study developed a RAG2 blastocyst complementation strategy to specifically deplete CR2^+^ B cells in mice, leaving CR2^+^ FDC intact, which decreased T-dependent antibody responses elicited by a hapten-carrier conjugate [46]. In a subsequent study [62], immunizing with lower immunogen doses revealed detectable early antibody responses, antigen-specific germinal centers, enhanced somatic hypermutation, and increased antibody affinity. Despite preserving these response characteristics, antibody longevity and long-lived antibody-secreting cells in the bone marrow were profoundly reduced. This study used a canonical hapten-carrier immunogen in alum, an unlikely representative of immunogen/adjuvant combinations in modern vaccine development. Similar studies in CR2 knockout mice confirmed and extended these results using the bacteriophage Qβ as a model viral immunogen [48]. Immunization of wildtype mice with Qβ without adjuvant elicits [65, 66] strong and durable T-dependent antibody responses, providing a good model for multivalent viral nanoparticle vaccines like those discussed above. CR2^−^ mice immunized with a single dose of Qβ maintained normal early antibody responses and germinal center reactions but with abrogated antibody persistence. There was also a profound loss of Qβ-specific long-lived bone marrow plasma cells expressing Blimp-1, XBP-1, and Bcl-2, transcription factors required for terminal plasma cell differentiation. These studies reveal a strong dependence of durable antibody responses and long-lived plasma cells in the bone marrow on CD21^+^ B cells. We will return to the role of CD21 in regulating antibody responses to the envelope glycoprotein after discussing immune response dynamics and plasma cell subsets in humans.

### 2.4. Dynamics and Cellular Basis of Long-lived Antibody Responses

**Fig. (3).**
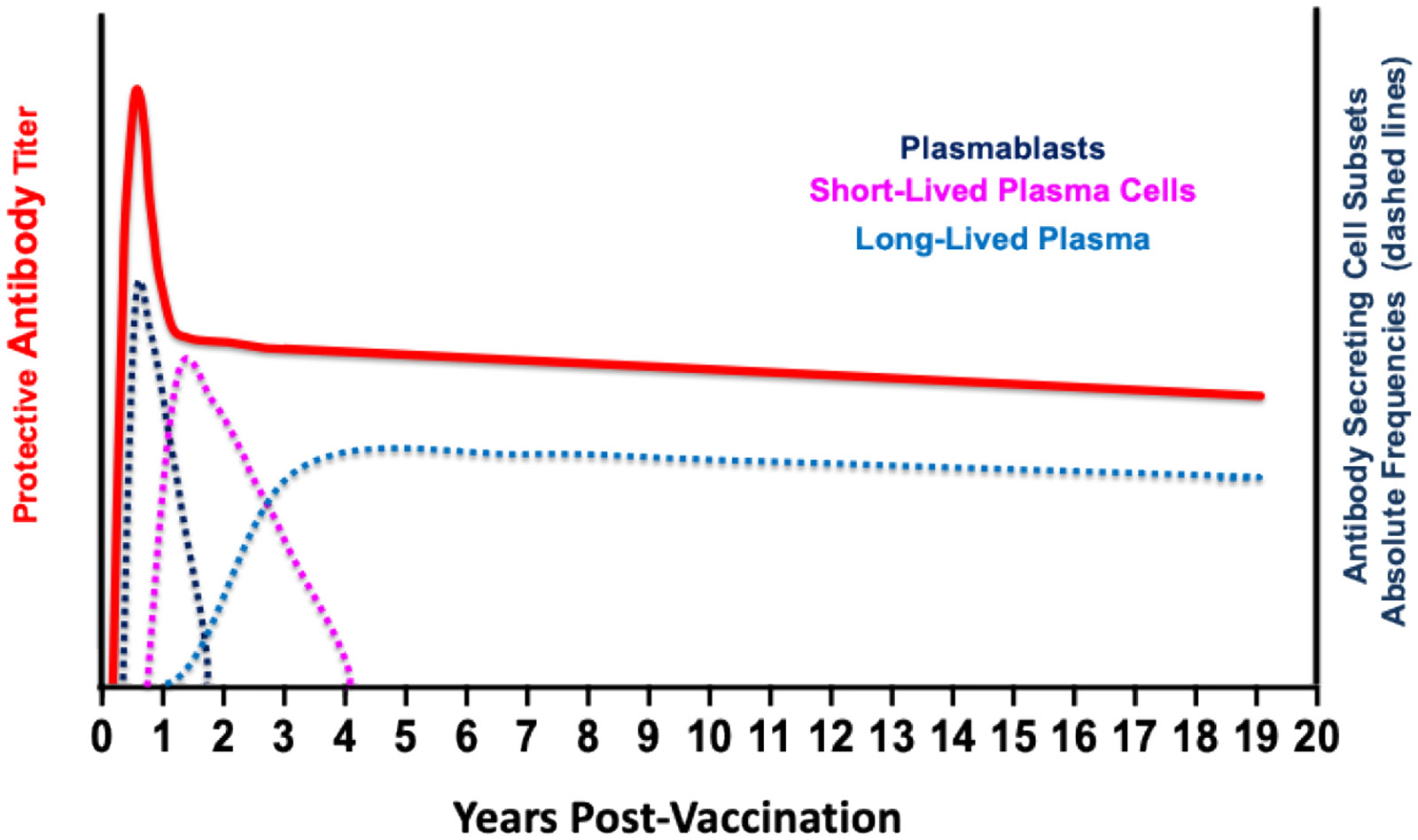
Dynamics of a Durable Antibody Response. Schematic depiction of response dynamics for a durable protective antibody response. The absolute frequencies of antibody-secreting cell populations illustrate the cellular sources of protective antibodies over time.

The dynamics of a durable antibody response are Figure 3 modified from our publication [16], based on the imprinted model of plasma cell lifespan model of Amanna and Slifka (c.f., Fig. **3** in [43]). Antibody response dynamics are characterized by three phases, each with a decay slope indicative of the cumulative contributions of distinct antibody-secreting cell (ASC) populations. These ASC populations are plasmablasts (PB), short-lived plasma cells (SLPC), and long-lived plasma cells (LLPC), which were reviewed in [16, 42, 43, 67, 68]. The first phase includes priming, peak antibody response, and the initial rapid decay of circulating antibodies. The priming phase can be infection, or one or more vaccinations separated by one to a few months. During this time, there is an exponential rise in circulating antibody titers that plateau and then decay with a steep initial slope. Circulating antibodies in the first phase are produced mainly by PB and SLPC in secondary lymphoid tissues, with plasma half-lives measured in days [42, 67, 69, 70]. Late in the first phase, LLPC begins seeding bone marrow niches, which continues into the second phase [71]. The second phase is characterized by an inflection point in the antibody decay curve, followed by the third phase, which is continuing decay. The rate of antibody decay in the third phase is the major determinant of antibody durability.

**Fig. (4).**
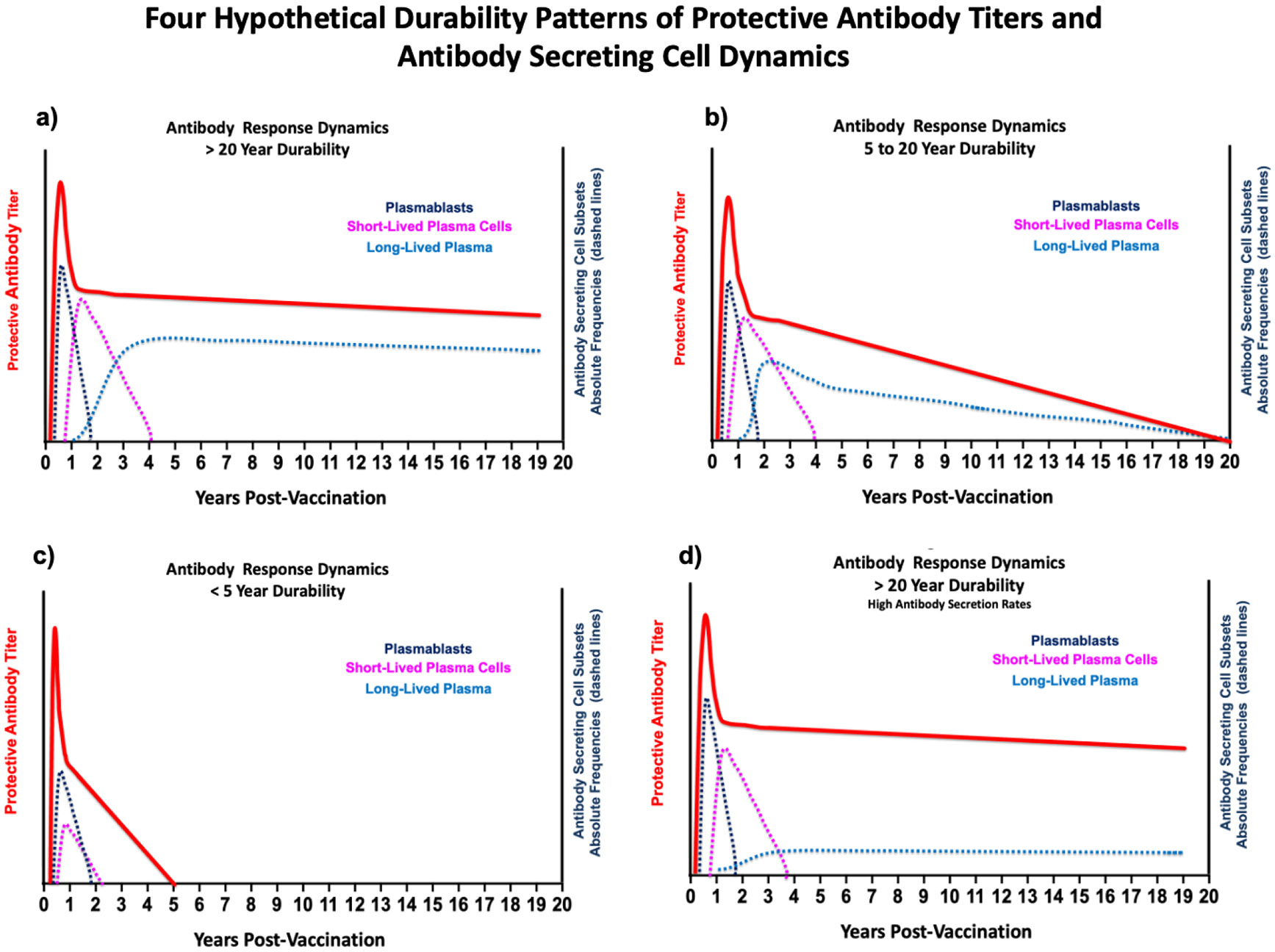
Antibody Response Dynamics for Four Different Durability Scenarios. Schematic depiction of response dynamics for protective antibody responses for four different durability scenarios. The absolute frequencies of antibody-secreting cell populations illustrate the cellular sources of protective antibodies over time. Panels **a**), **b**), and **c**) represent long-term antibody durability patterns influenced by decreasing frequencies (a>b>c) of long-lived plasma cells in the bone marrow. Panel **d**) represents the potential influence of high antibody secretion rates imprinted epigenetically on a low frequency of long-lived plasma cells in the bone marrow.

The sources of antibodies in the second phase are not well established. However, it is postulated that they were derived from PB and SLPC, generated from B_Mem_ activated by antigen depots and LLPCs. As antigen decays, the PB/SLPC response diminishes to the background, leading to the third phase, where the long-term antibody response is due solely to LLPCs. Studies in mice [72-76] and humans [77-79] have established that LLPC are the primary source of durable antibody responses in the third phase and are independent of persisting antigen and B_Mem_ [45, 52, 71, 72, 80-82]. It is also accepted that LLPC reside primarily in unique bone marrow niches that can be maintained without repeated antigen boosting [45,80-93]. These studies show that the generation of long-lived plasma cells is the primary determinant of durable antibody responses without repeated antigen exposure. This hypothesis predicts four scenarios of antibody-secreting cell dynamics that account for the three antibody durability patterns after vaccination discussed above. These scenarios are shown in Fig. (4).

As shown in Fig. (4), the early antibody titers after vaccination derive dynamically from three populations of antibody-secreting cells: plasmablasts, short-lived plasma cells, and long-lived plasma cells. Plasmablasts and short-lived plasma cells decay as antigen depots become depleted because these populations express surface immunoglobulin. After approximately three to four years, antibody titers are due solely to long-lived plasma cells in the bone marrow, determining whether the titers last > 20 years, 5 to 15 years, or < 5 years. These hypothetical time estimates and antibody titers shown in Fig. (4) are based on longitudinal vaccination studies discussed in our previous report [16]. As such, the panels in Fig. (4) are heuristic. Fig. (4) is also intentionally vague about the precise phenotypes of the antibody-secreting cell populations. More precise relationships require new studies to quantify each population over time for relation to antibody titers. Fortunately, recent studies, largely from the groups of Frances Lee and Inaki Sanz at Emory, are providing an increasingly clear picture of antibody-secreting cell heterogeneity in humans, with emphasis on bone marrow plasma cell subsets. Before turning to those studies, Figure 4d illustrates an additional level of complexity as it is possible to have high circulating levels of antibody with low frequencies of long-lived bone marrow plasma cells because there can be significant variations in antibody secretion rates, which are a key and understudied variable contributing to antibody durability.

### 2.5. The Importance of Determining Antibody Secretion Rates in Antibody Durability Studies

There is evidence that antibody secretion rates of individual B cells/plasma cells differ based on immunization conditions, both in vitro [94] and *in vivo* [95], possibly through epigenetically controlled B7-CD28 interactions [96, 97]. A recent study using a novel microdroplet system that enables massively parallel surveillance of antibody-secreting cells [98, 99] revealed secretion rates spanning approximately four logs for mice immunized with tetanus toxoid in Freund's adjuvant [100]. Importantly, these methods [98,99] are readily adaptable to quantification of antibody secreting rates of both peripheral and bone marrow antibody secreting subsets in humans, which will be key in future studies of antibody durability. However, there is only one key study in which antibody secretion rates by long-lived plasma cells in the bone marrow have been studied in the context of antibody durability [95]. Although conducted with less advanced techniques, this study [95] provides unique mechanistic insight into antibody durability that strongly implicates antibody secretion rates as a major, albeit largely ignored variable contributing to antibody durability. In this study, immunization with a T-independent pneumococcal polysaccharide induced significantly higher and more durable circulating antibody titers than a T-dependent form, despite lower frequencies of bone marrow plasma cells. The authors attributed this discrepancy to faster antibody secretion rates in the T-independent group, a distinction that underpins the antibody dynamics illustrated in Figure 4d. To our knowledge, this remains the only study to directly compare these parameters directly, underscoring the critical need to measure both plasma cell frequency and per-cell secretion rate when evaluating the mechanisms governing long-term antibody responses.

### 2.6. Antibody Secreting Cell Subsets in the Bone Marrow

Historically, studies of mice [45, 80, 81] and nonhuman primates [72, 101, 102] have convincingly shown that antibody-secreting cells in the bone marrow long after infection or immunization are long-lived. More recent studies in humans reveal significant heterogeneity of antibody-secreting cells in the bone marrow. A seminal study [103] identified four distinct plasma cell populations in human bone marrow, only one of which appeared to be long-lived. This was determined by sorting bone marrow antibody-secreting cells based on the expression of CD19, CD38, and CD138 into four populations (summarized in Table 1). Population D was identified as long lived because it was the only one in which antibody-secreting cells (by ELISPOT analysis) were found for measles and mumps, which the volunteers had not been exposed to for more than 40 years. Also, population D largely lacks surface immunoglobulin, which is consistent with the absence of CD19, and it has a distinct immunoglobulin heavy chain variable region (VH) repertoire that matches the circulating antibodies for measles and mumps, which is distinct from the other populations. Finally, population D has a unique morphology with condensed nuclei, a high cytoplasm to nucleus ratio, and autophagy vacuoles. These properties are consistent with many studies over the years showing that terminally differentiated plasma cells remain immobile in plasma cell niches where they are dedicated solely to the synthesis and secretion of antibodies, which can be as much as 30% of newly synthesized cytoplasmic protein [104].

Subsequent studies by the investigators identified elements of the bone marrow niche supporting long-lived plasma cell survival [105, 106]. They also showed that the generation of long-lived plasma cells is epigenetically imprinted [107] as predicted by Amanna and Slifka [43]. More recent studies [108] of bone marrow plasma cell populations defined a new maturation pathway as they mature to the long-lived population D. A maturational trajectory of early, transitional, and late antibody secreting cell signatures was defined, where population D predominantly exhibited the late signature. Only populations A and B exhibited the earlier signature, although they were also found in the transitional and late groups. These studies suggest a developmental relationship among the bone marrow plasma cell populations that finally lead to the long-lived population D. The authors previously described similar studies for peripheral plasma cell subsets, where five new subsets of antibody-secreting cells were defined using CD19, CD38, and CD138 and unique transcriptomes. Each of the subsets was involved in vaccine-specific responses and showed long-term survival potential in vitro. It will be important to determine which of these subsets seed the bone marrow and mature into the long-lived population D. These elegant studies provide a framework for future studies of antibody durability in humans and augur for further definition of plasma cell populations in mice and rhesus macaques, where comparable information is missing. The remaining discussion will focus on how the above considerations impact the development of a bnAb-based vaccine against HIV.

### 2.7. The Problem of Antibody Durability Confronting the Development of a bnAb-based HIV Vaccine

Despite over forty years of intensive efforts, a vaccine against HIV-1 remains elusive. Despite this caveat, it is now accepted that an ideal HIV-1 vaccine should block transmission of most, if not all, global viral variants and that broadly neutralizing antibodies (bnAbs) are the strongest candidate for protection. The ability of neutralizing antibodies to block HIV-1 transmission in people was established unequivocally in two recent Phase 2b efficacy trials of bnAb VRC01 to prevent HIV-1 acquisition in healthy volunteers [109]. While there was no overall efficacy in those trials, subgroup analyses enabled the important "proof of concept" hypothesis that strongly supports further development of bnAb-based vaccines and therapeutics. For example, the trial design included secondary analyses of the transmitting strains based on their ability to be neutralized by VRC01. When prevention efficacy was stratified into three categories based on neutralization sensitivity (IC_80_), efficacy was 75.4% in individuals whose viruses were neutralized by VRC01 at IC_80_ < 1ug/ml. By contrast, prevention efficacy was 4.2% and 3.3% for individuals whose viruses were neutralized by VRC01 at IC_80_ 1-3 ug/ml or IC_80_ >3 ug/ml, respectively, which resulted in an overall efficacy not greater than placebo. Subsequent analyses showed that serum-neutralizing titers of VRC01 are a biomarker of reduced HIV-1 acquisition [110, 111]. In agreement with prior passive immunization studies in nonhuman primates (NHP) [112], sustained bnAb concentrations approximately 200-fold greater than the serum dilution required for in vitro IC_80_ will have > 90% prevention efficacy for neutralization-sensitive, circulating viruses [110, 111]. These results frame two problems confronting antibody-based HIV-1 vaccine development. The first problem is the development of vaccine candidates that elicit bnAbs, which is being pursued intensively by multiple strategies (reviewed in [113-115]) where germline targeting approaches are starting to bear fruit in NHP [116-118] and people [119]. The second problem is how to maintain protective bnAb levels over time without continuous boosting. The importance of this problem is made clear by the two recent Phase 2b efficacy trials of bnAb VRC01 where acquisition was blocked in 75.4% of individuals who were exposed to viruses with neutralization sensitivity of <1ug/ml IC_80_. Given an HIV-1 vaccine that elicits the desired bnAb breadth, it would have to achieve and maintain titers of up to 200 μg/ml to be optimally effective. Thus, maintaining durable protective titers of antibodies after vaccination is the second major problem confronting the development of antibody-based vaccines against HIV-1.

The antibody durability problem is illustrated by a recent study from Dennis Burton's group showing that vaccine elicited protection of NHP against SHIV_BG505_ correlated with autologous neutralizing antibody titers of 1:500 IC_50_ in a pseudovirus assay[120]. Protection against six repeat, low-dose SHIV_BG505_ challenges was approximately 75% at eleven weeks post-challenge, waning to approximately 30% by twenty weeks after an additional six SHIV_BG505_ challenges beginning between weeks eleven and twelve post-immunization. Notably, this level of protection required four immunizations with an HIV-1_BG505_ SOSIP trimer in a potent adjuvant, which frames the acute need to increase the durability of protective antibody responses to HIV-1. This NHP result is also consistent with the effect of antibody durability on protection observed in the RV144 trial, which we have discussed previously [16]. Taken together, these results strongly support new studies to understand why protective antibody responses to the HIV-1 envelope glycoprotein wane rapidly over time and to develop new strategies to mitigate the problem, especially for bnAb-based HIV vaccines that are most likely to afford strong protection. Below, we develop the hypothesis that the HIV envelope glycoprotein does not reliably elicit CD21-dependent long-lived plasma cells in the bone marrow. Rather, the literature suggests that this glycoprotein and immunogens based on it elicit tissue resident T-bet^+^ CD21^low^ memory B cells that are epigenetically geared to produce large quantities of antibody when the antigen persists. Antigen dependence distinguishes this population from the long-lived CD21-dependent plasma cell population that produces antibody independently of persisting antigen.

### 2.8. Does the HIV Envelope Glycoprotein Elicit Long-lived Plasma Cells in the Bone Marrow?

During natural HIV infection, our group has documented a discordance between anti-gp120 antibodies in circulation and the memory B cell pool [121]. This study was carried out in our local cohort of PLWH, some of whom control viremia without anti-retroviral therapy for HIV [122]. Some of these PLWH have ongoing low-level viremia that correlated with neutralization breadth [123] and enables the isolation of near pan-neutralizing bnAbs against the CD4 binding site of Env [124]. Because we found anti-Env antibody specificities archived in the circulating B_Mem_ pool that were not detectable in circulation, we questioned whether HIV infection elicits long-lived anti-Env plasma cells in the bone marrow. The poor durability of anti-Env vaccine studies in nonhuman primates [120] and people [125, 126] discussed above is consistent with a deficit in long-lived anti-Env plasma cells in the bone marrow. In this regard, it is important to note that anti-Env antibodies in PLWH are predominantly produced by T-bet^+^ CD21^low^ memory B cells in both acute and chronic infection cohorts [127]. A more recent study extended this result, showing that Env-specific CD19^+^T-bet^+^ CD21^low^ memory B cells accumulate outside of lymph node germinal centers in viremic PLWH [128]. These findings are consistent with older studies of viremic PLWH reporting high frequencies of “atypical” CD21^low^ B cells with plasmacytoid morphologies that were categorized as “exhaustive” based on in vitro studies [129], which are characteristics of T-bet^+^ CD21^low^ B cells. T-bet^+^ CD21^low^ B cells are a unique population of largely tissue resident B_Mem_ that are programmed to rapidly differentiate into antibody-secreting cells in situations of chronic or repeated antigenic stimulation, including autoimmunity and infections, including HIV, malaria, COVID-19, and hepatitis (reviewed in [130-134]). It is important to note that recent studies point toward a unique pattern of early B cell activation as a major contributor to the development of neutralization breadth against HIV in an acute seroconversion cohort [135]. Interestingly, the activation phenotype favoring durability resembles that of T-bet^+^ CD21^low^ B cells, although this subset was not studied in [135].

Originally, it was thought that T-bet^+^ CD21^low^ B cells respond poorly to antigen in vitro, although they express surface immunoglobulin and CD19 [129]. More recently, it was found that T-bet^+^ CD21^low^ B cells respond better in vitro to membrane-associated antigen, whereas they responded poorly to the same antigen in solution [136]. As with HIV Env specific antibody responses in PLWH (discussed above [127, 128]) is notable that anti-influenza hemagglutinin and anti-malaria CSP antibody responses also derive largely from T-bet^+^ CD21^low^ B cells in both infection [137, 138] and vaccination [139], where the vaccination studies are particularly informative. Vaccination with seasonal influenza vaccines elicited newly derived T-bet CD21^low^ B cells specific for hemagglutinin [140, 141], whereas long-term memory B cell responses were maintained by conventional T-bet^low^ CD19^+^ CD21^hi^ CD27^−^ resting memory B cells. Similar results were reported for volunteers immunized with the CSP RTS, S malaria vaccine [142]. These vaccines share the property of the HIV Env vaccines discussed above; they do not elicit long-lived antibody responses. Since the T-bet^+^ CD21^low^ B cells specific for HIV Env, influenza hemagglutinin, and malaria CSP by definition lack CD21, we hypothesize that they will not generate long-lived plasma cells in the bone marrow as this is strictly dependent on the expression of CD21 on B cells in the mouse studies discussed above [46-48, 62, 63]. To our knowledge, this is the first time a potential connection has been made between the poor durability of responses to HIV Env, influenza hemagglutinin, CSP vaccines, and CD21, which modulates BCR signaling strength. This hypothesis is also prompted by studies of bone marrow plasma cells specific for Env in HIV infection and hemagglutinin in volunteers after immunization with seasonal influenza vaccines.

Studies in viremic PLWH showed that anti-Env responses are largely made by CD19^+^CD3^hi^CD138^hi^ bone marrow plasma cells [143], which corresponds to the short-lived population B defined in [103]. Population B may be a precursor to population D, but it is not long-lived [108]. To our knowledge, there have been no other studies of Env-specific bone marrow plasma cell subsets in PLWH or uninfected people vaccinated with the Env-based HIV vaccines. However, several groups, including ours, have such studies planned. In contrast to HIV, a study of bone marrow plasma cells after seasonal influenza vaccination is informative [144].

As pointed out above, seasonal influenza vaccination induced newly derived T-bet^+^ CD21^low^ B cells specific for hemagglutinin [140,141], whereas long-term memory B cell responses were maintained by conventional T-bet^low^ CD19^+^ CD21^hi^CD27^−^ resting memory B cells. These studies were restricted to circulating B_Mem_ cells. Another study [144] showed that recent hemagglutinin-specific bone marrow plasma cells (defined as CD138^hi^) arriving in the bone marrow shortly after immunization are short-lived, waning within one year after seasonal influenza vaccination. A smaller population of hemagglutinin-specific plasma cells was present before boosting, which remained stable throughout the study. These data suggest that bone marrow plasma cells elicited by influenza arise from distinct populations of B_Mem_. The early appearance of T-bet^+^ CD21^low^ B_Mem_ in the circulation after seasonal influenza vaccination suggests that they might be the source of the short-lived bone marrow plasma cells specific for influenza. It is also possible that the bone marrow plasma cell phenotypes are determined by whether they were elicited by influenza infection, where the hemagglutinin is multivalent on the virion surface, versus the typical seasonal influenza vaccine, where the membrane organization of hemagglutinin is disrupted by detergent, as proposed by Slifka and Amanna [42]. The seasonal influenza vaccines might also selectively stimulate T-bet^+^ CD21^low^ B cells because detergent disruption could increase the costimulation of TLR7 by viral RNA, favoring this subset's activation. These studies suggest that acute infection in a naïve host elicits classical long-lived plasma cells in the bone marrow, whereas repeated boosting or persistent infection skews the response to T-bet^+^ CD21^low^ B cells, which persist in tissues to provide long term antibody production in response to antigen in inflammatory environments (reviewed in [130-134]).

This conclusion is supported by studies in mice using the classical lymphocytic choriomeningitis virus (LCMV) model (reviewed in [145-147]). Infection of adult mice with the Armstrong strain of LCMV establishes an acute infection that is cleared immunologically, leading to durable anti-viral antibody production by bone marrow LLPC [45, 72, 73, 84]. By contrast, infection with the clone 13 LCMV variant establishes a chronic infection in mice [148]. This system has been a prototype for studying immunological differences between acute and chronic viral infections (reviewed in [147,149-151]). Several recent studies showed that acute LCMV infection elicits significant, but clonally restricted anti-LCMV antibody repertories as compared with chronic infections, where many new LCMV specific B cell clones emerge over time [73,152-156], which correlates with the predominance of T-bet^+^ CD21^low^ LCMV specific B cells in chronic LCMV infections [153,157]. Thus, the clonal dynamics of antibody specificities observed in influenza infections and vaccination in humans are also observed in the acute and chronic infection models of LCMV, where T-bet^+^ CD21^low^ B cells are responsible for the increased clonal diversity during the chronic phase. These studies provide a backdrop for thinking about whether this model extends to HIV infection and vaccination.

At this juncture, it appears that current HIV vaccines elicit protective antibody responses of short duration in both people [126] and nonhuman primates [120], implicating poor induction of bone marrow LLPC. By contrast, in chronically infected PLWH, most of the ongoing anti-Env antibody responses are provided by short-lived CD19^+^CD3^hi^CD138^hi^ bone marrow plasma cells [143] that arise from T-bet^+^ CD21^low^ B cells [127]. We have recent data in nonhuman primates that immunization with a monomeric conformationally constrained Env vaccine formulated with a TLR-4 adjuvant elicits very low levels of non-persisting, vaccine-specific antibody-secreting cells in the bone marrow, which is consistent with the HIV vaccine studies discussed above. By contrast, vaccination with an Env trimer formulated with a TLR7/8 agonist elicited high frequencies of anti-Env-secreting plasma cells in the bone marrow that persisted long after vaccination [102]. Unfortunately, the phenotypes of bone marrow plasma cells are unknown in nonhuman primates, in contrast to humans [103]. The most straightforward interpretation is that using a potent TLR7/8 adjuvant and a trimeric Env immunogen elicited responses in T-bet^+^CD21^low^ B cells responsible for the persistent antibody-secreting cells in the bone marrow. Persistent antibody-secreting cells were also observed in lymph nodes of the vaccinated nonhuman primates, which is consistent with the possibility that the Env-TLR7/8 agonist combination stimulates continuous activation of T-bet^+^CD21^low^ B cells that seed as plasma cells to the bone marrow. Interestingly, another study from the same investigators using the TLR7/8 adjuvant and a monomeric SARS-CoV-2 RBD vaccine elicited marginal frequencies of antigen-specific bone marrow plasma cells [158]. These studies show that the use of even the potent TLR7/8 adjuvant does not reliably ensure long-lived antibody responses via bone marrow plasma cells when the antigen is monomeric. Collectively, the above studies suggest that the identification of multimeric Env immunogen formulated with an adjuvant that selectively addresses B cells that differentiate into long-lived plasma cells via the CD21-dependent pathway will elicit more durable protective bnAb responses long after vaccination. Future studies are required to understand the dynamics of bone marrow plasma cell subsets in PLWH and after vaccination with candidate bnAb-based HIV vaccines. Two recent studies of spike-specific bone marrow plasma cell subsets after COVID-19 infection [159] or vaccination against COVID-19 [160] inform future studies of HIV-specific plasma cell dynamics.

### 2.9. Absence of Spike Specific LLPC in the Bone Marrow after Recovery from COVID-19 and after Vaccination Against COVID-19 with an mRNA Vaccine

The first study was carried out by our group using specimens from twenty healthy volunteers with a history of COVID-19 infection confirmed by polymerase chain reaction (PCR) or antigen tests, thirty percent of whom experienced severe COVID-19. All volunteers were infected during the first wave of COVID-19 in the United States, from March to November 2020, before the emergence of other variants and before vaccines were available. This study provides a unique, cross-sectional picture of anti-spike antibody responses occurring during the first wave of infection that would be difficult today due to the high prevalence of COVID-19.

Using the criteria for bone marrow plasma cell populations defined in [103] and summarized in Table 1, we evaluated ELISPOTs for total IgG, anti-tetanus IgG, and anti-spike IgG isolated from bone marrow aspirates by flow sorting [159]. We found approximately equal frequencies of anti-tetanus and anti-spike antibody secreting cells in the short-lived CD19^+^CD38^hi^CD138^hi^ population B. By contrast, anti-spike antibody secreting cells were largely absent from the long-lived CD19^−^CD38^hi^CD138^hi^ population D. The frequencies of anti-tetanus antibody secreting cells were approximately the same for both populations. Multivariate regression analysis showed that circulating anti-tetanus titers correlated with the frequency of anti-tetanus antibody-secreting cells in population D. By contrast, there was no correlation between circulating anti-spike antibodies and the frequency of antibody-secreting cells in this population, suggesting a deficient generation of spike-specific LLPC in the bone marrow after infection in volunteers in the first wave of COVID-19 infection. Building on previous modeling of influenza antibody durability [161, 162], we developed a mathematical model to predict the different outcomes for anti-spike and anti-tetanus LLPC in the bone marrow. The model is based on the competition of antigen-driven development of short-lived and long-lived plasma cells in the bone marrow and successfully recreates the long and short durability patterns illustrated in Fig. (4). The reader is referred to [159] for details of the mathematical model, which we will use in future studies to understand the differential basis for antibody durability elicited by vaccines.

The second study, from the Lee group at Emory, was a longitudinal analysis of plasma cell dynamics in healthy volunteers after mRNA vaccination [160]. The study enrolled 19 healthy adults who had received a total of two to five doses of SARS-CoV-2 mRNA vaccines. Each volunteer had also been boosted with the seasonal influenza vaccine within 1-12 months of the bone marrow aspirate and had completed the childhood series of the tetanus toxoid vaccine, with recent boosters ranging from 1 month to 24 years before the bone marrow aspirates. Five volunteers reported previous SARS-CoV-2 infections. Bone marrow aspirates were collected from all volunteers .5 to 21 months post-vaccination, one volunteer provided three bone marrow aspirates over 21 months, and another provided two aspirates over 6 months. Serum antibody responses and antibody-secreting cells specific for influenza, tetanus, and SARS-CoV-2 were quantified for each volunteer, where circulating antibody-secreting cells were measured 6-7 days post-vaccination, and bone marrow antibody-secreting cells were evaluated .5 to 21 months post the last mRNA vaccination against SARS-CoV-2. It was found that while influenza- and tetanus-specific ASCs in the bone marrow were readily detected in the long-lived population D, the SARS-CoV-2-specific antibody-secreting cells were largely confined to the short-lived populations A (CD19^+^CD38^hi^CD138^low^) and B (CD19^+^CD38^hi^CD138^hi^). Serum antibody responses to the SARS-CoV-2 spike protein waned rapidly within 3 to 6 months post-vaccination, which correlates with the low frequencies of anti-spike antibody-secreting cells in the long-lived population D.

Taken together, these two recent reports strongly suggest a deficit of spike-specific long-lived plasma cells in the bone marrow after infection with SARS-CoV-2 [159] or mRNA vaccination against this virus [160]. Both studies provide independent clues about why reinfections with coronaviruses occur so rapidly after infection. While antigenic drift is a known factor in the frequent SARS-CoV-2 reinfections, the paucity of spike-spike specific long-lived plasma cells in the bone marrow is also a likely factor. The relative contributions of antigenic drift and infrequent anti-spike LLPCs to rapid seasonal coronavirus infections are not known, but they are likely to contribute to this phenomenon. These studies also point toward undefined properties of the spike protein, as well as the microenvironments of infection or mRNA vaccination are responsible for the deficient generation of spike-specific LLPC. Identifying these factors will be key for understanding the poor durability of protective antibodies against SARS-CoV-2 and perhaps against other viruses.

## CONCLUSIONS

Achieving durable antibody-mediated protection remains a central goal in vaccine development, particularly for viral diseases such as COVID-19 and HIV, where protective immune responses are typically short-lived. The development of vaccines that can elicit long-term, high-affinity antibody responses without the need for frequent boosting would represent a transformative advance in global health. Currently, there is no reliable way to predict whether a given vaccine formulation will induce antibody responses that persist for more than five years post-vaccination. This limitation underscores the urgent need for systematic and mechanistic studies aimed at deciphering the cellular and molecular underpinnings of antibody durability. An ideal vaccine would establish lifelong protective immunity with a single or limited number of doses, avoiding the logistical and immunological challenges associated with repeated boosting.

As discussed throughout this review, one of the key variables influencing antibody durability is epitope multivalency—the presentation of multiple copies of an epitope within a single immunogen or immunogen complex. Multivalency can enhance B cell receptor (BCR) cross-linking, amplify B cell activation, and drive more robust germinal center (GC) responses, all of which contribute to the generation and maintenance of long-lived plasma cells (LLPCs) in the bone marrow. However, epitope multivalency, while beneficial, is not an absolute requirement for achieving durable antibody responses. For example, diphtheria toxin is a small dimeric protein [163], yet the inactivated diphtheria vaccine elicits antibody responses with a durability half-life of 19 years [11]. Similarly, tetanus toxin is monomeric [164], and the inactivated vaccine elicits antibody responses with a durability half-life of 11 years [11]. Thus, even monomeric or dimeric vaccines can elicit antibody responses with durability half-lives greater than 10 years, although it is possible that oligomeric forms of these immunogens with increased epitope multivalency might drive increased antibody durability. Regardless of the abilities of some paucivalent vaccines, others, such as the current spike mRNA COVID-19 vaccines, elicit very short-lived responses [160]. Understanding the mechanisms underlying these differences; however, the fact that all of the vaccines that protect for greater than 20 years all have high epitope multivalences (c.f. Table 1 in [18]), it is safe to assume that high epitope multivalency should be the default in approaches to increase antibody durability.

It is very likely that epitope multivalency also favors CD21-dependent BCR costimulation, which is essential for eliciting bone marrow LLPC and antibody durability in mice [46-48, 62, 63]. One of these studies [47] showed that epitope multivalency and CD21-mediated BCR costimulation interact to favor antibody durability. Higher epitope multivalency was required to elicit antibody responses without CD21, consistent with the BCR signaling strength hypothesis discussed above. Surprisingly, there have been no recent studies of CD21 and antibody durability, so these observations are limited to a few studies in mice, with the last published almost two decades ago [48]. We hope that the above discussion prompts renewed interest in the role of CD21 in antibody durability using newer mouse models for evaluating bnAb-based vaccines against HIV [165]. These models could also be valuable for screening immunogen-adjuvant combinations for their ability to elicit bone marrow LLPC and antibody durability in these studies.

In the discussions above, we have said little about the role of adjuvants in antibody durability, mainly because there are no comprehensive comparative studies of the abilities of different adjuvants to elicit truly durable antibody responses. Doing such a study is complicated by intellectual property barriers and the need to follow vaccinated volunteers for at least 5 years post-vaccination, preferably longer, to determine antibody durability accurately. Without such trials, we suggest that preclinical studies of candidate vaccine-adjuvant formulations include analyses of bone marrow LLPC and their antibody secretion rates throughout the study. This information is necessary to develop robust mathematical models that can predict antibody durability without carrying out extended studies. The weak immunogenicity of HIV Env vaccines has been discussed in the past [16,68,166], but there is no clear path forward as the problem is multifactorial. The most promising bnAb-based HIV Env vaccine candidates are trimers, often presented as multivalent nanoparticles, with only modest improvements in immunogenicity (reviewed in [68]). There is evidence that the high-mannose glycan shield of HIV Env dampens its immunogenicity [167-170], so it is possible that tailoring Env glycans might increase immunogenicity, as reported recently [171]. Future studies are required to determine whether these glycan modifications increase antibody durability. Against this backdrop, a trimeric Env immunogen formulated with a TLR7/8 adjuvant unambiguously increased antibody durability in nonhuman primates in step with anti-Env antibody-secreting cells in the bone marrow [102]. However, these antibody-secreting cells precise phenotype is unknown because the bone marrow plasma cell subsets have not been defined based on their lifespans in nonhuman primates. So, the anti-Env plasma cells in the bone marrow might be T-bet^+^ CD21^low^ antibody-secreting cells rather than the nonhuman primate equivalent of the human CD19^−^CD38^hi^CD138^hi^ population D LLPC. If this were the case, continuous antigenic stimulation would be required for long-term anti-Env antibody production, as shown recently in nonhuman primates using a long-prime, slow-delivery immunization strategy for an HIV Env trimer immunogen [172]. Because anti-Env antibodies in PLWH are derived predominantly from T-bet^+^ CD21^low^ antibody-secreting cells [127], it will be important in future studies to determine whether this holds for Env vaccination. It will also be essential to determine the relationship between Env-specific T-bet^+^ CD21^low^ and CD19^−^CD38^hi^CD138^hi^ bone marrow plasma cell populations, as well as the dependence of the latter on CD21 costimulation.

In closing, achieving durable antibody-mediated protection remains a critical objective in the rational design of vaccines, especially against challenging viral pathogens like HIV and SARS-CoV-2. Despite impressive advances in rapid vaccine development—as demonstrated during the COVID-19 pandemic—the ability to induce long-lasting antibody responses without repeated boosting remains elusive for many antigens. The discussion presented in this review emphasizes the multifactorial nature of antibody durability, highlighting the importance of immunogen multivalency, CD21-dependent B cell costimulation, adjuvant effects, glycan engineering, and bone marrow LLPC formation. These insights suggest that a combination approach will be necessary: one that optimizes antigen design, enhances innate and adaptive immune activation, and prioritizes long-term plasma cell survival in the bone marrow. Ultimately, the development of vaccines capable of providing durable, lifelong immunity will require a deeper understanding of the immunological mechanisms described herein, supported by innovative tools and collaborative long-term research initiatives. Meeting this challenge will not only improve protection against existing viral threats but also prepare for more effective responses to future pandemics and emerging infectious diseases.

## Figures and Tables

**Table 1.** Bone Marrow Plasma Cell Subsets.

Population A	Population B	Population C	Population D
CD19^+^CD38^hi^CD138^low^	CD19^+^CD38^hi^CD138^hi^	CD19^−^CD38^hi^CD138^low^	CD19^−^CD38^hi^CD138^hi^
Surface Ig^+++/−^	Surface Ig^++/−^	Surface Ig^+/−^	Surface Ig^−/−^
Typical PC Morphology	Typical PC Morphology	Typical PC Morphology	PC Morphology + Autophagy Vacuoles

**Note:** Principal characteristics of bone marrow plasma cell subsets as defined in [101].

## LIST OF ABBREVIATION

ASC: Antibody-Secreting Cells
BCR: B Cell Antigen Receptor
LLPCs: Long-lived Plasma Cells
EBV: Epstein-Barr Virus
CR2: Complement Receptor 2
SLPC: Short-lived Plasma Cells
PB: Plasmablasts
LCMV: Lymphocytic Choriomeningitis Virus
